# Decoding genomic rearrangements for cancer driver discovery

**DOI:** 10.1016/j.mocell.2025.100272

**Published:** 2025-08-30

**Authors:** Enyoung Seo, Sooyeon Park, Inho Park, Jinhyuk Bhin

**Affiliations:** 1 Graduate School of Medical Science, Brain Korea 21 Project, Yonsei University College of Medicine, Seoul 03722, Republic of Korea; 2 Department of Biomedical Sciences, Gangnam Severance Hospital, Yonsei University College of Medicine, Seoul 03722, Republic of Korea; 3 Department of Pathology and Center for Precision Medicine, Gangnam Severance Hospital, Yonsei University College of Medicine, Seoul 03722, Republic of Korea; 4 Department of Biomedical Systems Informatics, Yonsei University College of Medicine, Seoul 03722, Republic of Korea

**Keywords:** Genomic rearrangements, Noncanonical rearrangements, Whole-genome sequencing

## Abstract

Somatically acquired genomic rearrangements are common genomic alterations that contribute to malignancy by altering the expression or activity of cancer-related genes in human cancer. Genomic rearrangements play a crucial role in tumor development by contributing to driver events in approximately 25% of cancer patients. Most rearrangements are nonrecurrent and lack functional impact. However, some rearrangements produce functional transcripts and act as cancer drivers that may be therapeutic targets. The growing availability of whole-genome and matched RNA-sequencing data from large patient cohorts offers tremendous opportunities to identify novel, clinically relevant drivers arising from genomic rearrangements. In this review, we summarize current knowledge of driver rearrangements as therapeutic targets and highlight recent discoveries of functional transcripts such as intergenic fusions generated by noncanonical rearrangements. We also discuss computational approaches to decode rearrangement patterns and leverage large-scale whole-genome data to discover novel drivers.

## INTRODUCTION

Next-generation sequencing technologies have revolutionized cancer research over the past decade by enabling high-throughput, cost-effective profiling of tumor genomes. Early large-scale initiatives, such as The Cancer Genome Atlas (TCGA), pioneered the systematic characterization of somatic mutations across multiple cancer types (Bailey et al., 2018). These efforts extensively cataloged driver mutations and their prevalence, serving as an unprecedented resource to discover and validate novel oncogenic variants with potential clinical utility. However, TCGA studies primarily relied on whole-exome sequencing with limited information on genomic rearrangements formed through other mechanisms, including DNA replication errors, repair defects, and genomic instability.

Genomic rearrangements are large-scale structural changes in the genome, which include deletions, insertions, duplications, inversions, translocations, and more complex events, such as fold-back inversions, chromoplexy, and chromothripsis (Cosenza et al., 2022). These rearrangements are also distinguished into canonical types, which produce in-frame fusions between 2 known coding genes through well-characterized mechanisms, and noncanonical types, which involve atypical breakpoints such as intergenic regions, antisense orientation, complex multistep rearrangements, or regulatory element repositioning (Vellichirammal et al., 2020, Voronina et al., 2020, Yin et al., 2022). Notably, genomic rearrangements can alter local copy numbers by introducing extra copies or deleting genomic segments around breakpoints (Li et al., 2020b). In cancer, somatically acquired rearrangements usually affect cancer genes by disrupting gene structure, generating functional gene fusions, or inducing copy number variations. Whole-genome sequencing (WGS) in large cancer cohorts has become increasingly feasible due to reduced sequencing costs and it enables comprehensive characterization of genomic rearrangements. Notable consortia, such as the International Cancer Genome Consortium, the Pan-Cancer Analysis of Whole Genomes, and the UK 100,000 Genome Project, revealed different patterns of genomic rearrangement and exposed novel oncogenic drivers (Aaltonen et al., 2020, Zhang et al., 2019, Murugaesu et al., 2022). The International Cancer Genome Consortium’s UK breast cancer project sequenced 560 whole genomes to reveal 6 structural variant (SV) signatures and potential driver rearrangements that directly affect cancer-related genes (Nik-Zainal et al., 2016). As a follow-up to this work, the Pan-Cancer Analysis of Whole Genomes consortium analyzed 2,600 primary cancers across 38 tumor types to expand the number of SV signatures from 6 to 16 (Li et al., 2020b). Cancer patients harbor an average of 4.6 somatic driver variants that include 1.3 driver rearrangements, which suggests their substantial contribution to tumor development (Aaltonen et al., 2020). More recently, the UK 100,000 Genome Project performed WGS on 2,023 colorectal cancers to provide a detailed landscape of driver variants with hotspot rearrangements that contribute to tumorigenesis (Cornish et al., 2024). Therefore, there have been substantial advancements in the identification of oncogenic alterations arising from genomic rearrangements due to recent large-scale WGS analyses.

Our review outlines the current landscape of precision medicine that targets oncogenic rearrangements. The review also emphasizes the clinical relevance of both canonical and noncanonical genomic rearrangements with potential therapeutic implications. While discussing the underlying sources of genomic rearrangements, we elaborate on the genomic features that result in the formation of SVs. Finally, we introduce the different computational approaches that can identify signals of positive selection against a nonrandom background shaped by these features.

## PRECISION THERAPEUTICS FOR GENOMIC REARRANGEMENTS IN CLINICAL ONCOLOGY

As of 2024, according to OncoKB Level 1/2, which includes Food and Drug Administration (FDA)-recognized biomarkers (Level 1) and National Comprehensive Cancer Network guideline-based standard-of-care biomarkers (Level 2), there are approved targeted therapies for 12 types of genomic rearrangements in clinical oncology: *ABL1*, *ROS1*, *NTRK1/2/3*, *RET, ALK*, *FGFR1/2/3*, *PDGFRA/B*, *KMT2A*, *RARA*, *BRAF*, *NRG1*, and *JAK2* (Table 1). Collectively, 29 FDA-approved drugs target these rearrangements. Notably, these clinical rearrangements result in gene fusions that produce chimeric transcripts. Most involve kinase genes that generate kinase-domain-preserving, in-frame fusions that drive constitutive kinase activation. In addition to rearrangements, these kinases also harbor hotspot mutations or gene amplifications, suggesting multiple mechanisms of oncogenic activation (Stransky et al., 2014, Zehir et al., 2017). However, not all alterations confer equal clinical importance. This underscores the need to understand the functional significance of distinct oncogenic events for precision oncology (Berger and Mardis, 2018, Drilon et al., 2017, Katoh, 2019).

**Table 1:** List of genomic rearrangements targeted by FDA-approved or standard-of-care therapies

Rearrangement	Disease	Drug name	Approved test	Partner genes	Other mutations
ABL1	CML	Bosutinib, Imatinib, Nilotinib, Dasatinib, and Ponatinib	FISH RT-PCR	5′-BCR (90%)	T315I
ROS1	NSCLC	Crizotinib, Entrectinib, Repotrectinib, Ceritinib, and Lorlatinib	FISH RT-qPCR Targeted DNA-seq IHC	5′-CD74 (38-54%) 5′-EZR (13-24%) 5′-SDC4 (13-24%) 5′-SLC34A2 (5-10%)	
NTRK1/2/3	NSCLC, THCA, and solid tumors	Entrectinib, Larotrectinib, and Repotrectinib	FISH Targeted DNA-seq RT-PCR Targeted RNA-seq	5′-LMNA (for NTRK1) 5′-ETV6 (for NTRK2/3) 5′-BTBD1 (for NTRK3)	
RET	Solid tumors	Selpercatinib, Pralsetinib, and Cabozantinib	FISH Targeted DNA-seq RT-PCR	5′-NOA4 (33%) 5′-CCDC6 (30%) 5′-KIF5B (6%)	C611Y, C618R, S891A, C630R, C609Y, V804M, A883F, R886W, M918T, C634R, C620R, and A883T
ALK	NSCLC	Alectinib, Brigatinib, Ceritinib, Crizotinib, Ensartinib, and Lorlatinib	FISH Targeted DNA-seq Targeted RNA-seq IHC	5′-EML4 (93%) 5′-NPM1 5′-RANBP2	
FGFR2/3	CHOL, BLCA	Futibatinib, Pemigatinib, and Erdafitinib	FISH Targeted DNA-seq IHC	3′-BICC1 (for FGFR2) 3′-BICC1 (for FGFR2) 3′-ATE1 (for FGFR2) 3′-TACC3 (86%)	
PDGFRA/B	MDS/MPN	Imatinib	FISH RT-PCR	5′-FIP1L1 (for PDGFRA) 5′-EBF1 (for PDGFRB) 5′-KANK1 (for PDGFRB) 5′-ETV6 (for PDGFRB)	
KMT2A	AML/ALL	Revumenib	RT-PCR Targeted DNA-seq	3′-MLLT1 (19%) 3′-MLLT3 (12%)	
RARA	APL	ATRA/ATO	FISH RT-PCR	5′-PML (95%)	

ALCL, anaplastic large-cell lymphoma; ALL, acute lymphoblastic leukemia; AML, acute myeloid leukemia; APL, acute promyelocytic leukemia; B-ALL, B-cell acute lymphoblastic leukemia; BLCA, bladder carcinoma; CML, chronic myeloid leukemia; CEL, chronic eosinophilic leukemia; CHOL, cholangiocarcinoma; GIST, gastrointestinal stromal tumor; IMT, inflammatory myofibroblastic tumor; LGG, low-grade glioma; MDS, myelodysplastic neoplasms; M/LNeo, myeloid/lymphoid neoplasms with eosinophilia; MPN, myeloproliferative neoplasms; NSCLC, non–small-cell lung cancer; PA, pilocytic astrocytoma; PDAC, pancreatic adenocarcinoma; SKCM, skin cutaneous melanoma; THCA, thyroid carcinoma; ATO, arsenic trioxide; ATRA, all-trans retinoic acid; FISH, fluorescence in situ hybridization; IHC, immunohistochemistry; qPCR, quantitative polymerase chain reaction; RT-PCR, reverse transcription polymerase chain reaction.

Twelve types of genomic rearrangements targeted by FDA-approved or standard-of-care therapies were obtained from OncoKB. The OncoKB Level 1 (FDA-approved drugs targeting FDA-recognized biomarkers) and Level 2 (FDA-approved drugs targeting standard-of-care biomarkers) annotations are used in the table.

The specificity of fusion partners varies significantly among rearrangements. Some are highly partner-restricted, eg, *ABL1* that exclusively forms *BCR-ABL1* fusions in chronic myeloid leukemia is treated with kinase inhibitors including Imatinib and Dasatinib (Massimino et al., 2020). *RARA* fusion, which is predominantly involved in *PML-RARA* fusion in acute promyelocytic leukemia, responds to all-trans retinoic acid and arsenic trioxide (Zhang et al., 2021). However, rare *RARA* fusions, such as *PLZF-RARA*, *NPM1-RARA*, and *TFG-RARA* fusions, which conserve the DNA-binding domain and ligand-binding capacity, vary in their responses to these therapies (Zhang et al., 2021). In bladder cancer, *FGFR3* rearrangements predominantly involve *TACC3* as they occur primarily through tandem duplications due to their genomic proximity (Ascione et al., 2023). This prevalence suggests selective oncogenic advantages conferred specifically by the *FGFR3-TACC3* fusion (Costa et al., 2016).

However, some clinically targetable rearrangements exhibit notable fusion partner promiscuity, such as *FGFR2*, *ALK*, and *NTRK1/2/3* (De Luca et al., 2020, Shreenivas et al., 2023, Westphalen et al., 2021). This suggests that oncogenicity primarily stems from intrinsic kinase activation rather than the fusion partner because these rearrangements fuse with diverse genomic loci, including intergenic regions. A recent study revealed that loss of exon 18 (E18), rather than specific fusion partners, is the key determinant of the oncogenic potency of *FGFR2* (Zingg et al., 2022). E18-lacking *FGFR2* fusions exhibit stronger tumorigenic potential than full-length fusions. This contrasts with *FGFR3*, where fusions occur predominantly with *TACC3*, suggesting fundamental mechanistic differences in oncogenicity between the 2 receptors (Krook et al., 2021). Similarly, *ALK* and *NTRK* rearrangements occur with various nonspecific partners and reinforce the therapeutic focus on targeting the kinase domains (Chen et al., 2021, Dai et al., 2022). Irrespective of specific partner identity, FDA-approved inhibitors ensure broad applicability by directly targeting kinase domains across various fusion contexts. Therefore, kinase inhibition is a central strategy in precision oncology to treat cancers driven by genomic rearrangements.

## ONCOGENIC NONCANONICAL INTERGENIC REARRANGEMENTS IN CANCER

Traditional gene fusion studies have overlooked many rearrangements involving intergenic regions and mainly focused on rearrangements between protein-coding genes (Gao et al., 2018, Liu et al., 2025). However, recent studies have highlighted the significance of rearrangements beyond the gene body in cancer progression and treatment (Yun et al., 2020). The WGS analysis of 268 TCGA tumors showed that 62% (166/268) of rearrangements involved at least 1 intergenic breakpoint to reveal the prevalence of intergenic rearrangements (Yun et al., 2020). Many clinical cases of intergenic rearrangements in targetable kinase genes, such as *ALK*, *RET*, and *ROS1*, which showed good response to tyrosine kinase inhibitors, were identified through target panel sequencing (Cai et al., 2021, Li et al., 2020a, Paratala et al., 2018, Shlien et al., 2016, Yao et al., 2022, Zhai et al., 2025, Liao et al., 2022). Similarly, in a targeted sequencing analysis of 30,450 lung cancer patients, kinase fusions were identified in 3,411 patients, of which 538 (16%) of the 3,411 patients harbored intergenic rearrangements (Yao et al., 2022). In total, 624 kinase-intergenic rearrangements were detected in 538 patients. The most frequent kinase-intergenic rearrangement was *ALK* (117/624, 19%), followed by *EGFR* (54/624, 9%), *RET* (40/624, 6%), *ERBB2* (37/624, 6%), and *ROS1* (26/624, 4%). Of these, 316 were clinically targetable, suggesting the production of functional proteins with preserved kinase activity, and most of these (219/316, 69.3%) retained the kinase domain. Notably, 3 out of 6 patients with intergenic-*ALK/ROS1* rearrangements responded favorably to Crizotinib, implying their therapeutic relevance (Yao et al., 2022).

**Fig. 1:**
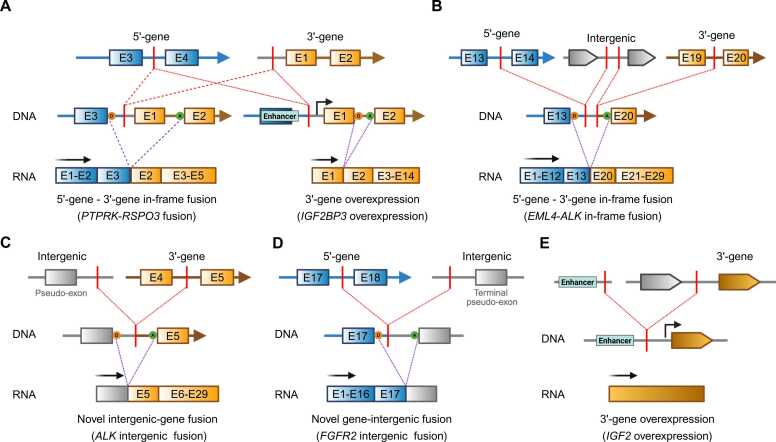
Scenarios of noncanonical intergenic rearrangements forming functional transcripts. (A) Intergenic rearrangements resulting in either a canonical in-frame fusion transcript (left) or overexpression of an intact mRNA from a 3′-gene via enhancer hijacking (right). These events are often driven by a breakpoint occurring in the upstream intergenic region of the 3′-gene. Only partial exon structures are shown for both the 5′-gene (blue boxes and arrow) and the 3′-gene (yellow boxes and arrow) in the DNA. Gray line in the upstream of the 3′-gene denotes intergenic regions. Yellow circles labeled “D” and green circles labeled “A” in DNA indicate splice donor and splice acceptor sites, respectively. Red bars represent rearrangement breakpoint loci in the DNA. Red edges link breakpoint pairs forming the rearranged DNA structure, while purple edges connect exons in the resulting mRNA. Black arrows above RNA structures indicate the direction of transcription. “E” in the gene box indicates an exon. (B) Rearrangements involving multiple intergenic breakpoints resulting in a canonical in-frame fusion transcript. Gray arrow boxes represent genes located near an intergenic breakpoint. (C) Intergenic rearrangements resulting in a fusion transcript consisting of an intergenic sequence–derived pseudo-exon containing an upstream start codon (gray box) and exons following a breakpoint in the 3′-gene. (D) Intergenic rearrangements resulting in a fusion transcript consisting of exons upstream of the breakpoint in the 5′-gene and a pseudo-exon derived from the intergenic sequence containing a stop codon (gray box). (E) Intergenic rearrangements resulting in gene overexpression through enhancer hijacking, in which a pseudo-enhancer derived from the intergenic sequence induces an overexpression of the 3′-gene.

The transcriptional consequences of kinase-intergenic rearrangements vary (Li et al., 2020a, Yun et al., 2020). In many cases especially when the rearrangement breakpoint occurs in the upstream region of the 3′-partner gene, canonical, in-frame fusion transcripts are produced through splicing events that skip intergenic sequences (Fig. 1A, left). This mechanism has been frequently observed in several known oncogenic fusions, such as *TMPRSS2-ETV4* and *TMPRSS2-ERG* in prostate cancer, *PTPRK-RSPO3* in colorectal cancer, and *TPM3-ROS1* in lung cancer (Yun et al., 2020). Alternatively, such rearrangements cause overexpression of the 3′-partner gene through enhancer hijacking (Fig. 1A, right). The overexpression of *IGF2BP3* in thyroid cancer has been attributed to this pattern that has been recurrently identified in rearrangements between the upstream intergenic region of *IGF2BP3* (3′-gene) and intragenic regions of *THADA* or *WARS* (5′-gene) (Yun et al., 2020). Kinase-intergenic rearrangements also involve multiple intergenic breakpoints depending on the complexity of the underlying rearrangement, as exemplified by *EML4-ALK* fusions (Li et al., 2020a) (Fig. 1B). The resulting chimeric transcripts can be translated into fusion proteins, which may represent effective targets for kinase inhibitors. Some kinase-intergenic rearrangements are not expressed or translated, which results in nonproductive alterations due to either the loss of the transcription start site, reading frame, and splice sites or due to degradation by nonsense-mediated decay. Several studies assessed the expression of fusion genes using targeted RNA-seq panels. However, such panels are limited in detecting fusions with unexpected partners or noncanonical breakpoints. Therefore, it is still unclear whether these kinase-intergenic rearrangements are truly unexpressed or simply missed due to the expression of noncanonical transcripts containing novel intergenic pseudo-exons. Novel pseudo-exons derived from intergenic sequences provide splicing acceptor or donor sequences that preserve reading frames or generate novel junctions and, thus, contribute to the formation of functional transcripts (Fig. 1C and D). A previous study reported an *ALK* rearrangement that involves a breakpoint in the intergenic region between *Linc00308* and *D21S2088E*, which results in the expression of a fusion transcript comprising a novel exon derived from the intergenic region and *ALK* exon 20 (Zhang et al., 2020) (Fig. 1C). ALK protein level was also confirmed with ALK D5F3 antibody that binds to an epitope in the C-terminal portion of the ALK protein. Notably, the patient with this fusion showed sensitivity to Crizotinib and proved that this noncanonical *ALK* rearrangement can be a therapeutically targetable variant (Zhang et al., 2020). Pan-cancer *FGFR2* analysis also revealed several forms of noncanonical rearrangements that conserve the kinase domain but lack E18. The loss of E18 can be induced not only by canonical gene-gene fusions but also by noncanonical fusions, such as out-of-frame, out-of-strand, and intergenic rearrangements (Zingg et al., 2022) (Fig. 1D). At the RNA level, some of these fusions show supporting evidence to suggest the expression of functional transcripts lacking E18. Importantly, pharmacogenomic datasets confirmed that the loss of *FGFR2* E18 is a clinically actionable biomarker for FGFR-targeted therapies (Zingg et al., 2022). Another example is the *UBE3C-*intergenic fusion, a C-terminal truncating gene-intergenic rearrangement that incorporates a terminal pseudo-exon derived from an intergenic sequence, which has been implicated in distal hereditary motor neuropathies (Cutrupi et al., 2023). Enhancer hijacking or promoter swapping can generate increased expression of the intact 3′-gene mRNA when a breakpoint occurs in the upstream intergenic region of the 5′-gene and another in the downstream intergenic region of the 3′-gene (Fig. 1E). Several cancer-associated genes, including *IGF2* in colorectal cancer, *ETV1* in prostate cancer, *IGF2BP3* in thyroid cancer, and *FUT5* in breast cancer, are upregulated through this mechanism (Yun et al., 2020).

Once not considered as relevant, noncanonical rearrangements are emerging as important drivers of cancer progression and therapeutic response. Genomic and transcriptomic studies increasingly show that these rearrangements can generate functional transcripts that preserve drug-targetable domains, underscoring their clinical relevance. These findings support the broad inclusion of noncanonical rearrangement events in diagnostic and therapeutic strategies, especially for kinase-driven cancers.

## GENOMIC FEATURES INFLUENCING THE FORMATION OF REARRANGEMENTS

Recent large-cohort WGS studies have facilitated systematic identification of oncogenic drivers mediated by genomic rearrangements (Rheinbay et al., 2020, Murugaesu et al., 2022). Similar to hotspot point mutations, the accumulation of recurrent breakpoints near or within cancer-related genes may be interpreted as a sign of positive selection for tumor evolution. These rearrangements can result in gene fusions, enhancer hijacking, or loss of regulatory domains to promote clonal expansion that enhances proliferation, survival, or therapy resistance (Yun et al., 2020, Zingg et al., 2022). Therefore, mapping breakpoint hotspots is pivotal to discover novel oncogenic drivers. Genomic rearrangements are nonrandomly distributed across the genome, as their occurrence is influenced by the local genetic contexts (Du et al., 2019, Kidd et al., 2010). Neutral events arising from intrinsic genomic instability rather than positive selection could be the source of many recurrent rearrangements. Correcting for such background biases is essential to accurately detect functional drivers.

**Fig. 2:**
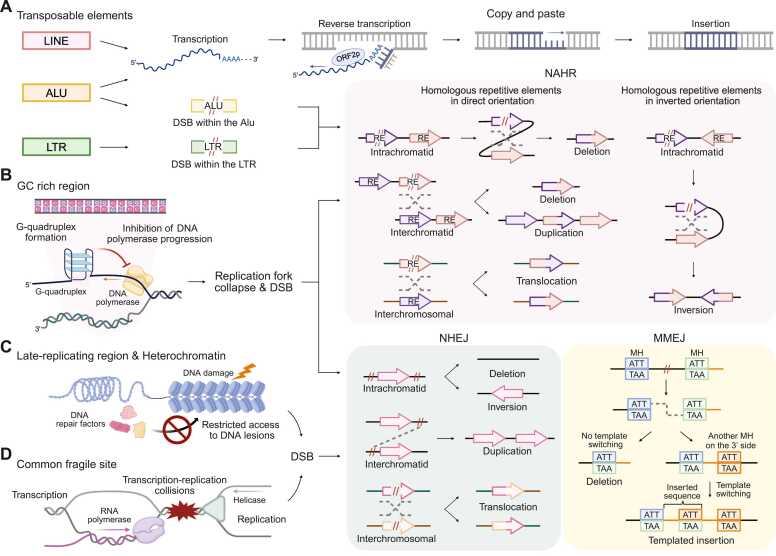
Mechanisms of formation of genomic rearrangements mediated by genomic covariates. (A) Rearrangements associated with repeat elements. LINE-1 elements transpose through a copy-and-paste mechanism using their own reverse transcriptase and endonuclease, whereas SINEs such as Alu elements hijack these functions from the LINE-1 open-reading frame 2 (ORF2) protein. Structural rearrangements are promoted by sequence homology between repetitive elements, such as Alu elements or LTRs, which facilitate NAHR. (B) Rearrangements associated with GC content. High GC regions form stable secondary structures such as G-quadruplexes that stall replication forks, leading to DSBs and NAHR-, NHEJ-, and MMEJ-mediated rearrangements. (C) Rearrangements associated with replication timing and chromatin status. Late-replicating regions, often enriched in heterochromatin, exhibit restricted accessibility to DNA repair machinery with increased reliance on error-prone NHEJ and MMEJ pathways. (D) Rearrangements associated with CFSs. Under replication stress, transcription at CFSs can interfere with ongoing DNA replication to cause replication-transcription collisions. These collisions stall replication forks and could trigger fork collapse, generating DSBs that are often repaired by error-prone NHEJ or MMEJ, thereby promoting rearrangement formation. Arrow boxes labeled “RE” in the NAHR-mediated rearrangement represent repeat elements. “MH” in the MMEJ pathway denotes microhomology. MH located in different regions is distinguished by box colors, and an example MH sequence (ATT/TAA) is used to illustrate the mechanism of MMEJ-mediated rearrangement. Orange lines indicate inserted sequences. The gray dotted line indicates a homologous recombination in NAHR, end-joining in NHEJ, and end-joining through annealing of complementary MH sequences in MMEJ. For interchromosomal rearrangements, the 2 chromosomes are represented with different line colors. CFS, common fragile sites; GC, guanine-cytosine; MMEJ, microhomology-mediated end-joining; NHEJ, nonhomologous end-joining.

Due to their potential to increase the frequency of DNA double-strand breaks (DSBs) and to promote erroneous recombination between nonallelic sites, repetitive elements, including long interspersed nuclear element (LINE), short interspersed nuclear element (SINE), and long terminal repeat (LTR), are major contributors to genomic instability (Fig. 2A). LINE-1 is a transposable element comprising approximately 17% of the human genome (Beck et al., 2011, Paul et al., 2025). In cancer, it is often reactivated due to the loss of epigenetic silencing or functional p53, causing widespread insertional mutagenesis and rearrangements through a copy-and-paste mechanism (Beck et al., 2011, Paul et al., 2025). SINEs, such as Alu elements, also promote insertional mutagenesis by hijacking the enzymatic machinery of LINE-1 (Ade et al., 2013). Diverse genomic rearrangements occur due to the high sequence homology of these repeat elements that facilitate nonallelic homologous recombination (NAHR) following DSBs (Kim et al., 2016). Since NAHR is a key mechanism that underlies the formation of various rearrangements, the widespread distribution of such repetitive elements makes them prominent contributors to genomic instability (Carvalho and Lupski, 2016, Chen et al., 2014). Like Alu elements, LTR elements also contribute to rearrangement through NAHR between dispersed copies (Trombetta et al., 2016). Consequently, these repeat sequences serve as natural hotspots for DNA breakage and recombination. Therefore, it is pivotal to account for these repeat sequences in the identification of driver rearrangements.

Another important determinant of genomic rearrangements is the nucleotide composition of DNA (Fig. 2B). Genomic regions with high guanine-cytosine (GC) content are often associated with NAHR because they are usually enriched in low-copy repeats and segmental duplications, which thereby increase susceptibility to rearrangement (Jurka et al., 2004, Meunier and Duret, 2004). Additionally, high GC regions can form stable secondary structures, such as G-quadruplexes, which can cause replication fork stalling and collapse (De Magis et al., 2019). When replication stress remains unresolved, cells may enter mitosis with unreplicated DNA to form DSBs (Wilhelm et al., 2020). These DSBs are often repaired by error-prone mechanisms such as nonhomologous end-joining (NHEJ) or microhomology-mediated end-joining (MMEJ) (Owens et al., 2019, Sandoval and Labhart, 2004). These pathways are rapidly activated without using homologous templates, they increase the chance of rearrangements (Schimmel et al., 2019).

Replication timing also significantly influences the formation of rearrangements. Late-replicating regions, which are often associated with condensed, transcriptionally inactive chromatin, are more susceptible to DSBs and subsequent rearrangements (Du et al., 2019) (Fig. 2C). The limited accessibility of DNA repair machinery and homologous templates in these regions favors the use of NHEJ or MMEJ pathways (Du et al., 2019, Fortuny and Polo, 2018). Interestingly, deletions are often observed in late-replicating regions, whereas tandem duplications and unbalanced translocations are found in early-replicating regions that are often associated with euchromatic regions (Donley and Thayer, 2013). Notably, the association between replication timing, chromatin state, and rearrangement patterns varies across cancer types, underlying the distinct rearrangement landscapes observed in different tumor types (Li et al., 2020b).

Common fragile sites (CFSs) are genomic regions prone to DSBs under replication stress, primarily due to late replication timing and paucity of replication origins. There is increased dependence on NHEJ and MMEJ pathways because DSBs at CFSs often occur in late S/G2 phase or during mitosis when homologous recombination is less active (Schwartz et al., 2005, Truong et al., 2013). CFSs are also susceptible to replication-transcription conflicts that can exacerbate replication stress and contribute to the formation of DSBs (Li and Wu, 2020) (Fig. 2D). Notably, CFSs may give rise to potentially pathogenic rearrangements in disease-associated genes, which suggests that CFSs contribute to genomic instability by shaping a landscape of rearrangements that encompass both neutral passenger and driver events (Mitsui et al., 2010).

Since genomic features inherently influence the formation of rearrangements in cancer-associated genes, background biases should be judiciously incorporated in statistical models to detect true oncogenic driver events among passenger events. It is pivotal to incorporate such features into background models to avoid misinterpreting passenger rearrangements as signals of positive selection.

## COMPUTATIONAL MODELS FOR DETECTING ONCOGENIC REARRANGEMENTS

**Fig. 3:**
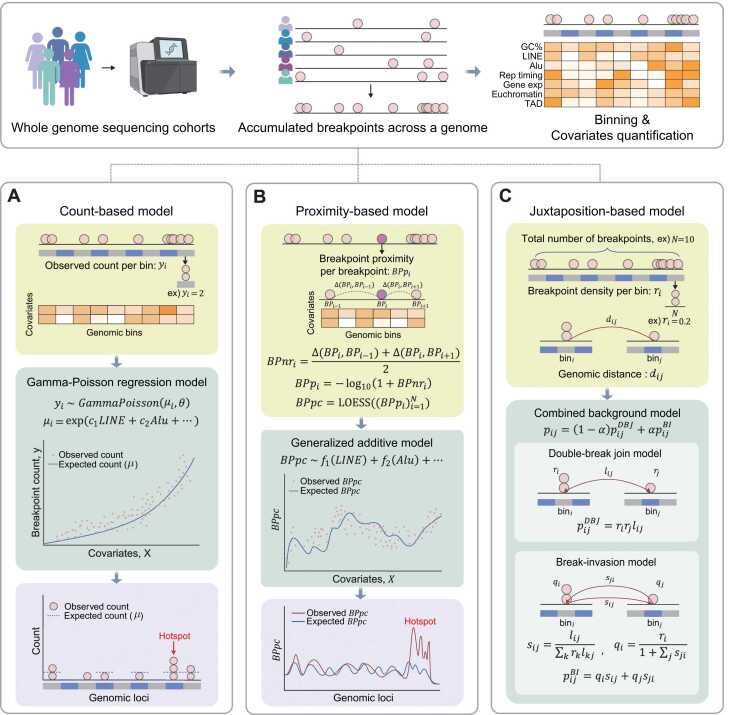
Computational approaches to identify breakpoint hotspot regions. Rearrangement breakpoints are identified using WGS data of a cancer cohort. Publicly available genomic covariates, such as GC content (GC%), LINE, Alu, replication timing (Rep timing), gene expression (Gene exp), chromatin states, and topologically associated domains (TAD), are quantified at a bin-level resolution. (A) Count-based model quantifies breakpoint accumulations at bin-level resolution. Gamma-Poisson regression is used to estimate expected breakpoint counts for bin *i* ($\mu_{i}$) and the dispersion θ by modeling the relationship between observed counts *y*_*i*_ and genomic covariates, with a coefficient *c*_*j*_ for each covariate *j*. Hotspots are defined as bins or segments with significantly higher observed breakpoints than expected. (B) Proximity-based model computes a proximity score (*BPp*_*i*_) for each breakpoint (*BP*_*i*_) as the −log₁₀ of its average distance to adjacent breakpoints. ∆ denotes the distance between breakpoints. Locally Estimated Scatterplot Smoothing (LOESS) then smooths these values to obtain *BPpc*. Covariates, assigned to each breakpoint using covariate-specific bins, are modeled against *BPpc* with a generalized additive model using covariate-specific smooth functions *f*_*j*_ to capture linear or nonlinear relationships. Hotspots are defined as regions that significantly deviate from the expected *BPpc*. (C) Juxtaposition-based model estimates the probability of background rearrangement between bins *i* and *j* ($p_{\mathrm{ij}})$ as a weighted sum of $p_{\mathrm{ij}}^{\mathrm{DBJ}}$ and $p_{\mathrm{ij}}^{\mathrm{BI}}$ components). In the double-break join model, $r_{i}$ and $r_{j}$ are computed as the number of breakpoints in each bin divided by the total breakpoint count *N*. $l_{\mathrm{ij}}$ denotes the fraction of all rearrangement pairs whose distances fall within the same interval as the distance between bins *i* and *j* ($d_{\mathrm{ij}}$). In the break-invasion model, $s_{\mathrm{ij}}$, derived from$\;l_{\mathrm{ij}}$, represents the conditional probability of a breakpoint at bin *i* invading bin *j*, while $s_{\mathrm{ji}}$ represents the reverse scenario. $q_{i}$ and $q_{j}$ are breakage probabilities that account for breakage events propagated from other bins. α is a weight parameter that controls the contribution of each model component.

To identify oncogenic drivers mediated by genomic rearrangements from cohort-based WGS, accurate modeling of the heterogeneous mutational background is required to distinguish true driver events from passenger alterations. Recent computational efforts based on machine-learning frameworks have attempted to model the distribution of background breakpoints by capturing relationships between genomic features and rearrangement breakpoint frequencies. These efforts can be categorized as: (1) count-based modeling, (2) proximity-based modeling, and (3) juxtaposition-based modeling (Fig. 3).

Count-based modeling segments the genome into fixed-size bins and quantifies breakpoint counts per bin from a cohort to estimate local breakpoint densities (Fig. 3A). This approach aims to model background breakpoint rates that vary across the genome by incorporating genomic covariates, such as GC content, repeat elements, replication timing, chromatin accessibility, and CpG/TpC ratios (Glodzik et al., 2017, Imielinski et al., 2017). One example is FishHook that employs a Gamma-Poisson regression framework to model bin-specific background breakpoint counts as a function of these covariates (Imielinski et al., 2017). The expected counts derived from this model serve as a null distribution to identify bins with a statistically significant number of breakpoints. Genomic regions containing such bins are considered potential hotspot regions that result from positive selection (Dubois et al., 2022, Rheinbay et al., 2020, Zhou et al., 2022). Since the results of this model depend on the fixed bin size, the model may miss hotspot regions that emerge at different binning resolutions. Piecewise constant fitting, a method frequently used to detect copy number alterations, may be further applied to segment the genome based on shifts in breakpoint density to enable more flexible hotspot detection (Glodzik et al., 2017, Nik-Zainal et al., 2016, Rustad et al., 2020, Cornish et al., 2024).

Breakpoint proximity–based modeling utilizes physical distances between breakpoints to estimate local breakpoint clustering (Fig. 3B). CSVDriver computes a breakpoint proximity curve (*BPpc*) based on a smoothed curve of breakpoint neighbor reachability (*BPnr*), which reflects the distances between breakpoints (Martínez-Fundichely et al., 2022). A generalized additive model is used to compute expected *BPpc* by modeling the nonlinear relationship between genomic covariates and *BPpc*. Significant breakpoint clusters are then identified by comparing observed vs expected *BPpc* values and computing a peak recurrence score to assess recurrence across samples (Martínez-Fundichely et al., 2022). This framework enables the discovery of hotspots without relying on fixed-size bins and allows for the modeling of both linear and nonlinear relationships with covariates.

Juxtaposition-based modeling focuses on identifying pairs of loci that are recurrently connected by genomic rearrangements rather than detecting breakpoint hotspots across samples within a cohort (Fig. 3C). SVSig-2D is a statistical framework that models the background frequency of rearrangements between bins *i* and *j* ($p_{\mathrm{ij}}$), which is computed as a weighted mixture of 2 models, double-break join and break-invasion models (Zhang et al., 2023). In the double-break join model, the rearrangement is assumed to arise from the fusion of 2 independently formed breakpoints, as seen in NHEJ or MMEJ. This model estimates the rearrangement probability ($p_{\mathrm{ij}}^{\mathrm{DBJ}}$) as the product of the breakpoint densities at bins *i* ($r_{i}$) and *j* ($r_{j}$), and a length factor ($l_{\mathrm{ij}}$) reflecting the distance between them. In contrast, the break-invasion model reflects NAHR-mediated rearrangements by assuming one breakpoint invades another. This model estimates the rearrangement probability ($p_{\mathrm{ij}}^{\mathrm{BI}}$) as the sum of 2 directional products: breakage probability at bin *i* ($q_{i}$) and the conditional probability of invasion into bin *j* ($s_{\mathrm{ij}}$), and vice versa. A binomial framework is used to statistically test observed breakpoint pairs against this background to identify significantly recurrent juxtaposition hotspots (Zhang et al., 2023). This provides a more comprehensive view of SV formation by considering both initial breakage and the rearrangement process. However, further refinement is needed to fully incorporate interacting factors such as DNA repair pathways, replication timing, and 3D genome architecture into a unified model that captures the underlying mechanisms of rearrangements.

## CONCLUSIONS AND FUTURE PERSPECTIVES

In the era of WGS for cancer genome profiling, the mutational processes, functions, and mechanisms of structural rearrangements have been widely explored. Ongoing large-cohort WGS projects, led by UK’s 100,000 Genome Project and the Hartwig Medical Foundation, together with combined multicohort analyses, are expected to further advance our understanding of driver alterations mediated by genomic rearrangements. Analyses of WGS integrated with matched transcriptome and/or mass-spectrometry-based proteomics data can facilitate the identification of functionally expressed drivers arising from noncanonical mechanisms, including pseudo-exon usage from intergenic regions, frameshift events, and antisense rearrangements. Recent advances in long-read DNA- and RNA-sequencing technologies are poised to accelerate the discovery and validation of these noncanonical drivers.

Significant challenges persist although the use of machine-learning frameworks to identify novel drivers through breakpoint distribution analysis is a promising approach. Detecting true signals of positive selection requires accurate modeling of the background distribution of breakpoint densities across the genome, which is typically predicted from genomic features. However, some of these features, such as replication timing, chromatin status, and topologically associated domains, vary within the same genomic regions depending on the cell and tissue types. This approach is limited in certain cancer types because these features are not available for all cell and tissue types. Therefore, expanding genomic feature data across diverse cellular and tissue contexts is crucial to improve the accuracy and applicability of machine-learning frameworks. Other challenges include the ambiguity in selecting an appropriate bin size for hotspot detection and the spatial correlations between adjacent bins, which are often simplistically assumed to be independent of each other in current machine-learning models.

Recent advances in deep learning models offer potential solutions to several of these challenges. Deep learning models have already been applied to structural variation research, particularly for improving SV calling (Cai et al., 2021, Lin et al., 2022, Liu et al., 2021) and genotyping (Linderman et al., 2024, Popic et al., 2023) by capturing complex, nonlinear patterns and discriminating true signals from noise in heterogeneous sequencing data. These capabilities could be leveraged to detect SV hotspots. For example, transformer-based models can learn long-range, multivariate interactions between nucleotide sequences and diverse genomic covariates (Choi and Lee, 2023). This enables inference of genomic contexts that predispose to SV formation. In addition, variational autoencoders can model the shared correlation structure of covariates into a latent space and identify anomalies as deviations from these learned patterns to provide a framework for hotspot discovery (Sado et al., 2024). Future advances in robust statistical modeling, powered by deep learning algorithms and expanding genomic feature datasets across diverse tissue types, will be critical to improve the identification of novel drivers.

## Author Contributions

**Sooyeon Park:** Writing – review & editing, Visualization. **Enyoung Seo:** Writing – review & editing, Writing – original draft, Visualization, Supervision. **Inho Park:** Writing – review & editing. **Jinhyuk Bhin:** Writing – review & editing, Writing – original draft, Visualization, Supervision, Funding acquisition, Conceptualization.

## Declaration of Competing Interests

The authors declare that they have no known competing financial interests or personal relationships that could have appeared to influence the work reported in this paper.
